# Case Report: First report of PIK3CA-related overgrowth spectrum revealed by refractory chylorrhea after breast-conserving surgery: failed thoracic-duct ligation

**DOI:** 10.3389/fsurg.2026.1820981

**Published:** 2026-06-23

**Authors:** Yue Wan, Peng Xu, Yu Zhang, Jing Xu, Baoxin Wang, Kaihua Zhao

**Affiliations:** 1 Department of Breast Surgery, Qingdao Central Hospital, University of Health and Rehabilitation Sciences, Qingdao, Shandong, China; 2 The Department of Quality Control, Qingdao Central Hospital, University of Health and Rehabilitation Sciences, Qingdao, Shandong, China; 3 Department of Pathology, Qingdao Central Hospital, University of Health and Rehabilitation Sciences, Qingdao, Shandong, China; 4 Department of General Surgery, Jiaozhou Maternity and Child Health and Family Planning Service Center, Qingdao, Shandong, China

**Keywords:** breast-conserving surgery, chylorrhea, PIK3CA, PIK3CA-related overgrowth spectrum, thoracic-duct ligation

## Abstract

**Introduction:**

Chylorrhea after breast-conserving surgery is exceedingly rare.

**Methods:**

We describe the case of a 38-year-old woman with refractory, isolated, cystic leakage from a breast cavity. All conventional interventions failed, whereas thoracic-duct ligation paradoxically increased chylous fluid drainage. Next-generation sequencing (NGS) of the extratumoral tissue revealed a somatic PIK3CA mutation, which led to a diagnosis of PIK3CA-Related Overgrowth Spectrum (PROS).

**Results:**

To our knowledge, this is the first reported case linking PROS to chylorrhea after breast-conserving surgery, aggravated by duct ligation.

**Discussion:**

Refractory surgical complications should be discussed and managed by a multidisciplinary team to identify or rule out occult diseases. These occult states that may be explained by somatic mosaic mutations are usually characterized by insidious or absent symptoms and are often diagnosed incidentally following surgical trauma or injury. In such contexts, precise genetic testing and targeted therapies may be pivotal and effective for diagnosis and treatment.

## 1 Introduction

Chylorrhea following breast-conserving surgery for cancer is a rare but challenging complication traditionally related to axillary lymph node dissection. Its pathogenesis may be linked to injury to great lymphatic trunks, including the thoracic duct (1). Thoracic-duct ligation is considered the most effective surgical treatment for cases refractory to conservative treatments, such as compression therapy and dietary change (2). However, treatment failure after conventional interventions or, more intriguingly, a paradoxical increase in leakage after the application of these interventions strongly indicates an underlying systemic or constitutive pathological process beyond a local anatomical injury.

PIK3CA-Related Overgrowth Spectrum (PROS) comprises heterogeneous mosaic disorders caused by activating somatic *PIK3CA* gene mutations, characterized by segmental overgrowth and intricate vascular or lymphatic anomalies (3, 4). PROS has substantial clinical heterogeneity, with potential involvement of soft tissues, bones, the central nervous system, and the vasculature. Lymphatic defects related to PROS, including lymphedema, lymphatic malformations, and chylous effusions, are increasingly being recognized (5). Constitutive activation of the PI3K-AKT-mTOR pathway is the pathophysiological foundation of PROS; this activation leads to inappropriate cell proliferation and survival, with consequent inappropriate lymphatic development and lymphatic dysfunction (6). This may produce a high-flow, architecturally disordered lymphatic network that functions erratically in response to conventional surgical occlusion of a single outflow vessel.

The case report describes a rare case of a patient with multifocal chylorrhea originating from a resection cavity after breast-conserving surgery without axillary dissection. Cautionary treatments were unproductive, and thoracic-duct ligation precipitated a sharp increase in chylous drainage. Ultimately, a PIK3CA mutation was detected by next-generation sequencing (NGS) of the resected mass and the surrounding skin tissues, confirming the diagnosis of PROS. This case challenges the conventional management paradigm and highlights the need for a multidimensional approach to refractory postoperative complications, as well as the critical role of refractory genetic testing in unmasking occult PROS and guiding subsequent therapeutic approaches.

## 2 Case description

### 2.1 Case report

A 38-year-old woman with a history of papillary thyroid carcinoma surgery 5 years prior, was diagnosed with breast cancer 1 week before her current admission. She subsequently underwent right-side breast-conserving surgery with sentinel lymph node biopsy. On postoperative day (POD) 2, the breast parenchyma cavity began draining 350 ± 50 mL/day of chylous fluid. A closed suction drain was inserted, and conservative management, including fasting, total parenteral nutrition, octreotide therapy, and negative pressure wound therapy, was initiated from POD 2 to POD 23 (3 weeks). Although the drainage fluid gradually cleared, the volume did not decrease. Re-exploration on approximately POD 23 revealed multiple foci of chylous leakage on the surface of the surgical cavity, with no axillary source identified. Indocyanine green (ICG) was injected into the interdigital webs of both feet, and fluorescence imaging was detected in the right breast cavity after 5 min (Figure 1). The leakage points were suture ligated, sealed with surgical glue, and managed with local compression bandaging. However, on POD 3 after re-exploration (approximately POD 26), the chyle leakage had returned to its preoperative state. An additional 3 weeks of conservative management (approximately POD 26 to POD 47) was unsuccessful. Thoracoscopic thoracic-duct ligation at the T9 level was performed on POD 47, during which intercostal lymphatic branches visualized by fluorescence were ligated. However, 2 days after this surgery (approximately POD 49), the volume of chylous drainage increased rapidly 800 ± 100 mL/day. Conservative management was continued for over 4 weeks (approximately POD 49 to POD 77) without substantial improvements. Given the patient's history of thyroid cancer surgery, potential compression or obstruction along the left venous angle could not be excluded. On POD 84, subsequent lipiodol lymphangiography was performed at another hospital, which showed that the thoracic duct was fully ligated at T9 with no major obstruction before its entry into the venous system. Periductal fibrolysis was performed at the venous angle; the volume of chylous drainage was maintained at 700–900 mL/day. Six days after undergoing periductal fibrolysis (90 days after the initial surgery), the drainage stopped, and the wound healed within a week (POD 94). The timeline is shown in Figure 2.

**Figure 1:**
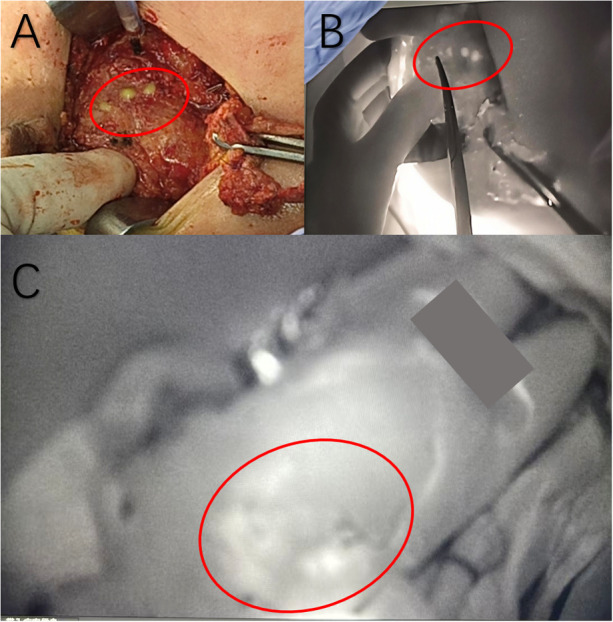
Chylorrhea from the right breast surgical cavity with fluorescence imaging and extensive right hemithorax fluorescence. **(A)** Chylorrhea on the inner wall of the surgical cavity. **(B)** Fluorescence imaging of the chylous leakage 5 min after pedal web space injection. **(C)** Extensive fluorescence imaging of the right hemithorax 10 min after pedal web space injection.

**Figure 2:**
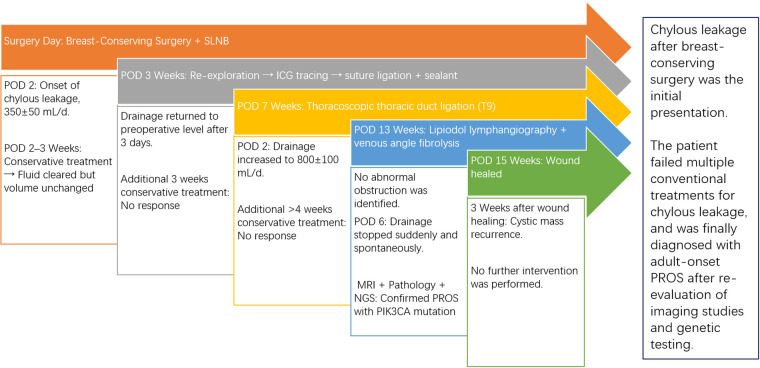
Diagnosis and treatment timeline.

Due to the unusual nature of these complications, the patient's imaging studies were reviewed in detail. T1-weighted, fat-suppressed, contrast-enhanced breast MRI revealed thickening of the right lateral chest wall with an infiltrative pattern, accompanied by prominent tortuous vascular dilation and multiple enlarged, enhancing lymph nodes (Figure 3). Pathologic examination of Hematoxylin and Eosin (HE) stained sections showed numerous dilated lymphatic vessels and intravascular thrombi, with abundant smooth muscle tissue observed within the lymph node structures (Figure 4). NGS of tissues from the resected surgical cavity identified the activating PIK3CA variant c.3140A > G (p.His1047Arg) at a 12.4% allele frequency, confirming low-level somatic mosaicism. Additionally, the tumor tissue also detected the PIK3CA mutation.

**Figure 3:**
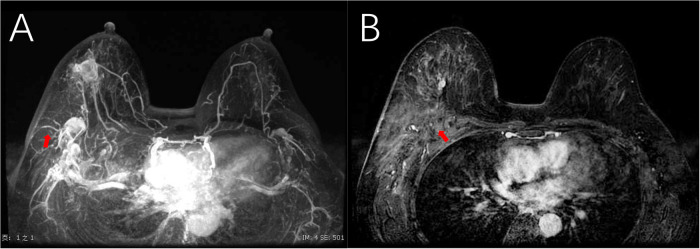
Breast contrast-enhanced MRI T1W1 fat-suppressed sequence. **(A)** Numerous dilated and tortuous vessels, together with markedly enlarged lymph nodes showing abnormal enhancement. **(B)** Markedly thickened chest wall and infiltrative tissue growth (Red arrowhead).

**Figure 4:**
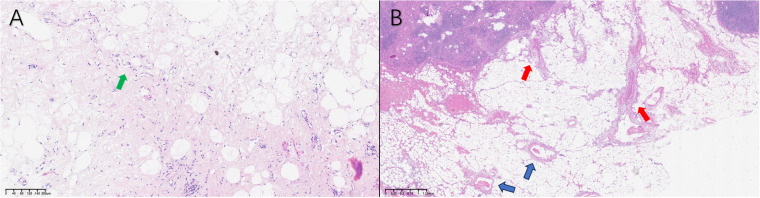
Histopathological images of the overgrown area (HE staining, 4 ×  magnification). **(A)** Numerous dilated, thin-walled lymphatic vessels filled with proteinaceous fluid (green arrow) in peritumoral tissue. **(B)** Multiple thrombus-containing vessels within the lymph node (blue arrow), and multifocal proliferating smooth muscle tissue (red arrow).

### 2.2 Follow-up

Three weeks after complete wound healing (approximately POD 115), a high-tension cystic mass reappeared in the surgical area. As no overt signs of inflammation or other substantial changes were observed in the incision or surrounding tissues during the subsequent 1-month follow-up, no further surgical intervention was undertaken. The patient received hypofractionated radiotherapy to the whole breast plus tumor bed, along with tamoxifen endocrine therapy on POD 160 after the initial surgery. She is recurrence-free at the time of this report, with good daily activity ability and satisfactory overall quality of life, and is satisfied with her current clinical outcome.

### 2.3 Diagnostic assessment

#### 2.3.1 Patient consent

This study adhered to the principles of the Declaration of Helsinki and was approved by the Ethics Committee of University of Health and Rehabilitation Sciences (Approval No.: KY202601701). The patient provided written informed consent for the collection of clinical data, publication, and use of accompanying images.

#### 2.3.2 Pathological section images

The surgically resected tissue specimens were fixed in 4% neutral buffered formalin, embedded in paraffin, and serially sectioned at a thickness of 4 μm for subsequent HE staining and immunohistochemistry (IHC) analysis. HE staining was performed following standard laboratory protocols to evaluate the sample's histological morphology. All sections were examined and imaged at 4 ×  magnification under a light microscope (Nikon Eclipse E100, Tokyo, Japan).

#### 2.3.3 Next-generation sequencing

Genomic analysis was performed using a targeted NGS assay for the *PIK3CA* gene. DNA was extracted from formalin-fixed, paraffin-embedded (FFPE) tissue of the resected breast cavity and surrounding skin using the RC1102 Nucleic Acid Extraction Kit (One Step Extraction for High Sensitivity FFPE Genomic DNA). Library preparation targeting the PIK3CA region was conducted, followed by sequencing. Bioinformatics analysis included alignment to the human reference genome (hg19). Somatic variant calling was focused on the PIK3CA locus.

## 3 Discussion

This case presents a clinical enigma with three paradoxical features. Rare chylorrhea originating from the breast cavity after surgery; increased drainage following thoracoscopic duct ligation, indicating retrograde chylous flow below the ligation site; and abnormal ICG lymphography kinetics with fluorescence appearing in the breast cavity within 5 min, much faster than the normal 60–120 min, indicating high-flow lymphatic channels.

Although prior cervical surgery may have altered lymphatic dynamics, it fails to fully account for the clinical picture. Imaging review revealed ipsilateral chest wall thickening and prominent vascular dilatation on MRI, and histology showed aberrantly increased and dilated lymphatics, multiple thrombi, and smooth muscle tissue ectopically extending into lymph nodes. These findings collectively shifted our focus from postsurgical injury toward an underlying developmental anomaly. Most critically, genetic analysis identified a somatic PIK3CA mutation, confirming the diagnosis of PROS.

Pathologically, the lymphatic phenotype of PROS, encompassing malformations, lymphedema, and chylous effusions, reflects a lymphatic network that is aberrantly dilated, hyperplastic, and hyperpermeable (7). We hypothesize that in our case, surgical trauma during breast-conserving surgery activated a pre-existing, occult, PROS-driven local lymphatic malformation, thereby creating a high-flow lymphatic leak. This pathophysiological model explains the condition's unresponsiveness to local surgical interventions, such as suture ligation and glue embolization. The markedly accelerated ICG transit, with fluorescence detected in the breast cavity within 5 min after injection, provides direct *in vivo* evidence of a high-flow state through structurally abnormal channels.

Thoracic-duct ligation is considered the definitive intervention for traumatic or postoperative chyle leakage, particularly when massive chyle leakage occurs after high axillary lymph node dissection on the left side, based on the assumption that the lymphatic drainage system is essentially normal (8). However, in the context of PROS, the aberrant lymphatics remodel downstream drainage. PROS results in a high-flow, architecturally disordered lymphatic network; ligation acutely raises intraluminal pressure and forces retrograde chyle extravasation. This concurs with the postoperative lipiodol lymphangiogram that confirmed unobstructed distal outflow despite intensified leakage after thoracic duct ligation, underscoring the need for a reassessment of lymphatic circulation before any further surgical intervention.

In this case, chyle leakage was confined to the surgical field of the left breast rather than the more common injury to the left subclavian thoracic duct; therefore, thoracic-duct ligation was not a recommended procedure. After prolonged conservative management and failed local interventions (suture ligation, glue sealing, and compression), the patient declined attempted ligation of the leaking lymphatic trunk due to the risk of incomplete closure. In the absence of more suitable options, multidisciplinary discussions led to an attempt at thoracic-duct ligation, which proved suboptimal. Upon subsequent review, we reasoned that if retrograde lymphangiography were available, it might have enabled precise localization and ligation of the leaking lymphatic trunk, representing a more rational and effective surgical approach, and this served as a valuable lesson.

The intensified chyle leak signaled retrograde collateral flow below the ligature (8), a paradox that can be traced to the pre-existing high-flow, dysplastic lymphatic network. The abrupt cessation of drainage on 6 days after lipiodol lymphangiography likely reflects a sterile, oil-induced inflammatory seal of the lymphatic breach, whereas the local cyst that reappeared after 3 weeks underscores the formidable force of aberrant lymphatic drainage. The mosaic PIK3CA variant (p.His1047R) that was eventually identified using targeted NGS confirmed a diagnosis of PROS (7). This diagnosis reinterprets the whole case: the failure and paradoxical blow-up following thoracic-duct ligation was not a mere coincidence but a foreseeable outcome of a general surgical rationality to this specific pathophysiology. This dual role of somatic PIK3CA mutation in tumor and stromal/lymphatic compartments creates a unique disease state in which breast cancer surgery can unmask otherwise clinically silent PROS, leading to atypical, refractory lymphatic complications such as high-flow chylorrhea (9).

Notably, when confronting atypical, treatment-resistant postoperative complications, particularly those involving the lymphatic or vascular systems, the clinical diagnostic approach should shift from pursuing further surgery to considering and investigating potential underlying systemic disorders, including PROS. We suggest early and focal genetic testing (e.g., low-pass, high-depth NGS) prior to repeat surgical intervention (10). Although debulking surgery for PROS may briefly provide symptomatic relief (11), the risk of short-term recurrence persists. However, recent research data strongly support the feasibility of molecular targeted therapies. For instance, mTOR inhibitors, such as sirolimus, can significantly reduce malformation volume and chyle output (12, 13), with approximately 60%–70% of patients achieving durable partial remission (14). Recently, a selective PI3K*α* inhibitor, alpelisib, has been proven to be effective in severe PROS phenotypes, including CLOVES syndrome and Klippel–Trenaunay syndrome (15). For patients with breast cancer complicated with PROS, targeted therapy yields dual clinical benefits: It controls PROS-induced chylorrhea while suppressing the proliferation of *PIK3CA*-mutated breast cancer cells (16). This synergistic effect suggests that perioperative targeted agents have the potential to prevent or treat refractory chylorrhea, thereby avoiding high-risk reoperation (17).

This case proposes a pathophysiological hypothesis that requires confirmation through additional patients and mechanistic studies. Our patient had estrogen-receptor-positive ductal carcinoma *in situ* and a PIK3CA mutation. PI3 K/AKT/mTOR signaling is known to promote tumor-cell proliferation and endocrine resistance (18). Whether the coexistence of PROS and the tumor PIK3CA variant influences the efficacy of adjuvant endocrine therapy or the long-term oncological prognosis of breast cancer remains unknown. Our patient remained disease-free for 3 months after radiotherapy and adjuvant endocrine therapy; however, extended follow-up was required to clarify any prognostic impact.

To our knowledge, this is the first study elaborating that breast surgery can trigger occult PROS; this was confirmed by somatic *PIK3CA* (p.His1047R) mutation, clinical imaging, pathological examinations, and genetic data. This case proposes a novel mechanism that surgical trauma activates congenital *PIK3CA*-driven lymphatic malformations and induces high-flow chylorrhea, explaining the failure of local conservative treatments. This report highlights the limitations of thoracic-duct ligation and advocates a precision strategy integrating genetic testing and targeted therapy. As a single-case report, it lacks large-cohort validation; therefore, long-term oncological prognosis and endocrine efficacy of concurrent PROS and *PIK3CA*-mutant breast cancer, as well as the value of perioperative PI3K/mTOR inhibitors, require further long-term follow-up and prospective trials.

## 4 Patient perspective

The patient stated that after undergoing breast-conserving surgery for cancer, she experienced refractory chylorrhea. Despite fasting, multiple re-explorations, and even thoracic duct ligation—which paradoxically increased drainage—she felt exhausted and confused. Fortunately, the medical team did not give up and ultimately diagnosed her with a rare condition, PROS, through genetic testing. Although a local cyst recurred three months post-surgery, she remains cancer-free after radiotherapy and endocrine therapy, with good daily function and satisfactory quality of life. She hopes her experience will help clinicians recognize the importance of investigating underlying genetic disorders when facing refractory postoperative complications.

## Data Availability

The original contributions presented in the study are included in the article/Supplementary Material, further inquiries can be directed to the corresponding authors.
